# A randomized trial of collaborative support for opioid taper after trauma hospitalization

**DOI:** 10.1186/s13011-024-00613-x

**Published:** 2024-06-24

**Authors:** Mark D. Sullivan, Laura Katers, Jin Wang, Sam Arbabi, David Tauben, Laura-Mae Baldwin

**Affiliations:** 1 Psychiatry and Behavioral Sciences, University of Washington, Seattle, WA 98195 USA; 2 Internal Medicine, Anesthesiology and Pain Medicine, University of Washington, Seattle, WA USA; 3 Harborview Injury Prevention and Research Center, University of Washington, Seattle, WA USA; 4 Harborview Trauma Surgery, University of Washington, Seattle, WA USA; 5 Family Medicine, University of Washington, Seattle, WA USA

**Keywords:** Collaborative care, Care management, Post-trauma care, Long-term opioid use, Chronic pain

## Abstract

The COTAT (Collaborative Opioid Taper After Trauma) Study was a randomized trial of an opioid taper support program using a physician assistant (PA) to provide pain and opioid treatment guidance to primary care providers assuming care for adult patients with moderate to severe trauma discharged from a Level I trauma center on opioid therapy. Patients were recruited, assessed, and randomized individually by a surgery research recruitment team one to two days prior to discharge to home. Participants randomized to the opioid taper support program were contacted by phone within a few days of discharge by the PA interventionist to confirm enrollment and their primary care provider (PCP). The intervention consisted of PA support as needed to the PCP concerning pain and opioid care at weeks 1, 2, 4, 8, 12, 16, and 20 after discharge or until the PCP office indicated they no longer needed support or the patient had tapered off opioids. The PA was supervised by a pain physician-psychiatrist, a family physician, and a trauma surgeon. Patients randomized to usual care received standard hospital discharge instructions and written information on managing opioid medications after discharge. Trial results were analyzed using repeated measures analysis. 37 participants were randomized to the intervention and 36 were randomized to usual care. The primary outcomes of the trial were pain, enjoyment, general activity (PEG score) and mean daily opioid dose at 3 and 6 months after hospital discharge. Treatment was unblinded but assessment was blinded. No significant differences in PEG or opioid outcomes were noted at either time point. Physical function at 3 and 6 months and pain interference at 6 months were significantly better in the usual care group. No significant harms of the intervention were noted. COVID-19 (corona virus 2019) limited recruitment of high-risk opioid tolerant subjects, and limited contact between the PA interventionist and the participants and the PCPs. Our opioid taper support program failed to improve opioid and pain outcomes, since both control and intervention groups tapered opioids and improved PEG scores after discharge. Future trials of post-trauma opioid taper support with populations at higher risk of persistent opioid use are needed. This trial is registered at clinicaltrials.gov under NCT04275258 19/02/2020. This trial was funded by a grant from the Centers for Disease Control and Prevention to the University of Washington Harborview Injury Prevention & Research Center (R49 CE003087, PI: Monica S. Vavilala, MD). The funder had no role in the analysis or interpretation of the data.

**Supplementary Information:**

The online version contains supplementary material available at 10.1186/s13011-024-00613-x.

## Introduction

The U.S. remains in the midst of an unprecedented opioid crisis. Nearly one million people have died since 1999 from a drug overdose. In 2023, an estimated 107,543 drug overdose deaths occurred in the United States. Opioids were involved in three-fourths of these drug overdose deaths and 92% of these opioid overdose deaths included synthetic opioids [1]. However, 30–50% of patients who develop OUD (Opioid Use Disorder) or die from opioid overdose still begin opioid use with prescribed opioids [2, 3]. There is also little evidence of the efficacy of long-term opioid therapy and growing evidence of harm, including impaired endogenous pain modulation and increased risk of self-harm [4, 5]. 

Opioid pain relievers are essential for treatment of pain after trauma, with over half of hospitalized trauma patients experiencing moderate to severe pain, and most patients still reporting pain at hospital discharge [4]. Persistent pain after trauma is common and associated with poor quality of life, psychological distress, reduced return to work, and the development of chronic pain [5–7]. These outcomes may arise from psychological trauma and post-traumatic stress disorder as well as the physical trauma itself [6]. Most serious trauma patients are discharged on opioids [8]. Patients discharged after major trauma are at high risk for opioid misuse and OUD, with two-thirds having at least one risk factor for unintentional opioid overdose and almost half showing signs of misuse [9]. It has been repeatedly observed that patients already on opioids at the time of their trauma or surgery are at higher risk for poor outcomes [7, 8]. 

Few opioid tapering guidelines exist for patients discharged after injury. Opioid tapering requires collaboration among the trauma center care team, the patient, and the PCP. This collaboration is especially difficult for patients living in rural areas remote from the trauma center, resulting in unequal risks for OUD and opioid overdose [13]. Small studies have begun to explore the value of transitional pain services for discharged trauma patients, but efficacy remains unclear [9]. We therefore conducted a pilot randomized clinical trial of an individualized opioid taper support program to support the PCPs of patients discharged from Level I inpatient trauma care after moderate to severe trauma and at high risk for prolonged opioid use because of the severity of their injury, their previous exposure to opioids, and their discharge to outlying counties in Washington State. We focused on support for PCPs rather than direct patient support because these clinicians have primary responsibility for pain and opioid care for discharged trauma patients and our study team had no direct clinical relationship with the patients. Our hypothesis was that: a 20-week collaborative pain care and opioid taper program will: (a) improve pain outcomes (pain severity, general activity interference, enjoyment of life interference) and (b) facilitate return to off opioids or pre-injury opioid dose, (c) improve secondary outcomes such as general patient-reported health status, and problem use of alcohol, cannabis, and illicit drugs.

## Methods

### Study design, participants and setting

**Fig. 1:**
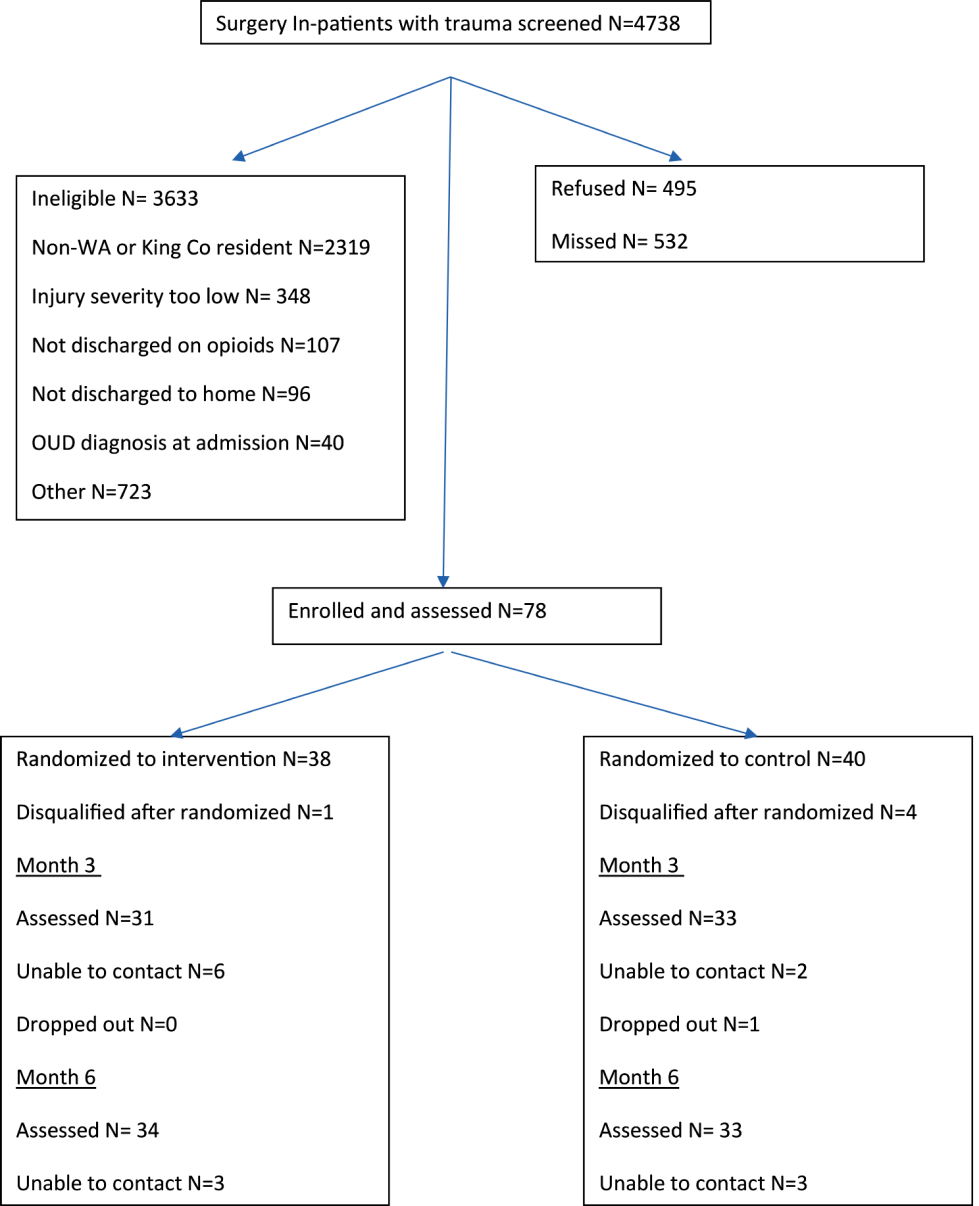
CONSORT Diagram for COTAT Study

This randomized controlled trial (RCT) was conducted at Harborview Medical Center in Seattle, Washington. Harborview Medical Center is the only Level I trauma center in the 5-state Northwest region of the US, covering 25% of the land mass of the US. This RCT used unblinded intervention administration but blinded outcome assessment. The study was approved by the UW institutional review board. All participants provided written informed consent. Study enrollment occurred from June 2020 and February 2022. Figure 1 shows participant flow through the study.

**Fig. 2:**
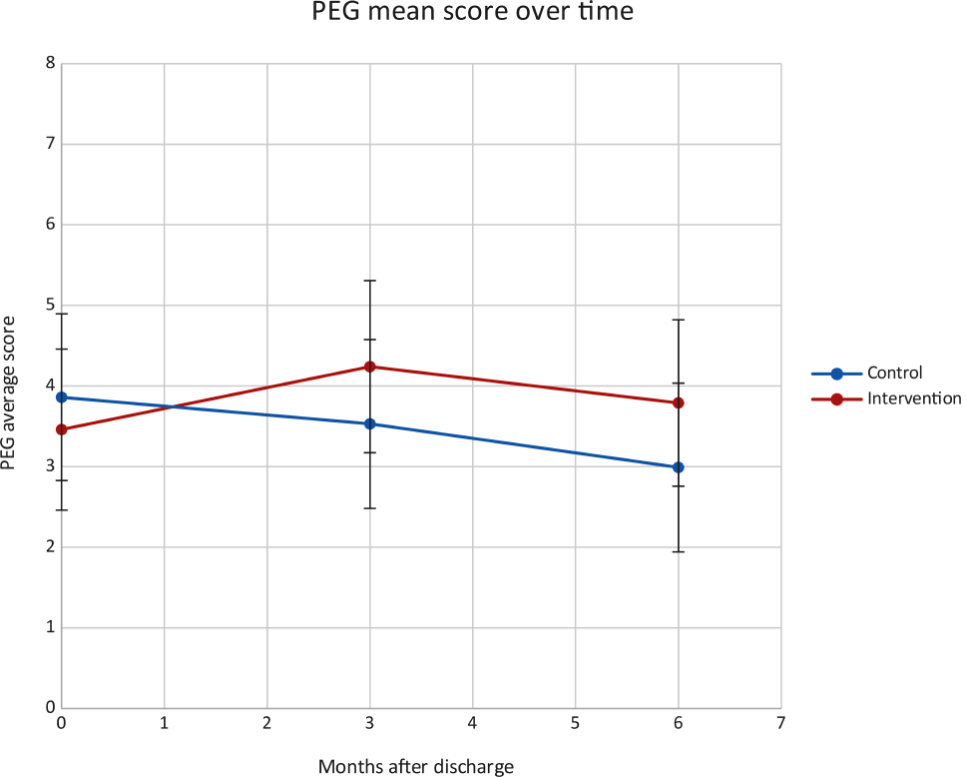
PEG mean score over time, intervention vs. control groups

This study sought to focus on trauma patients discharged on opioids who were at high risk for poor pain and opioid outcomes due to residence location and prior opioid exposure. Study inclusion criteria were: age *≥* 18 years, admitted to Harborview Medical Center after moderate or worse trauma (Injury Severity Score *≥* 4), speaks and reads English or Spanish, insurer in All Payer Claims Database (Medicare, Medicaid, other public WA insurance, WA commercial payors), planned to be discharged on opioids to Washington State counties outside King County. Study exclusion criteria included: admission Glasgow Coma Score < 15 (to limit effects of head trauma on intervention response and outcome assessment), unable to read English or Spanish, currently active cancer, enrollment in palliative care or hospice, plan for discharge to skilled nursing facility or assisted living, implanted device for pain control, OUD diagnosis in the electronic health record (including evidence of OUD treatment with buprenorphine, methadone, or naltrexone), use of illicit drugs in past month, psychotic symptoms, and psychiatric hospitalization or suicide attempt in past year. Patients with OUD were excluded from the study because opioid taper is not an appropriate treatment for most of these patients and a hospital program already exists to start these patients on buprenorphine maintenance prior to discharge.

### Procedures

Study participants’ electronic medical records were screened for eligibility during their hospital admission at Harborview Medical Center following moderate to severe trauma. Patients were recruited and consent obtained while inpatients by a surgery research recruitment team. Patients who provided consent were asked to complete a set of baseline questionnaires prior to randomization. Participants completed baseline questionnaires in the hospital prior to randomization. Follow-up assessments were conducted over the phone. Participants received $20 for completing the baseline assessment, $40 for the 12-week follow-up, and $50 for the 24-week follow-up. The CONSORT checklist for randomized trials was followed Supplemental Fig. 1).

### Intervention

#### Opioid taper support program

Though the taper support intervention was primarily aimed at supporting the PCP, it began with an introductory phone call from the PA interventionist to the patient within a few days of hospital discharge in order to confirm patient enrollment, clarify their PCP and follow-up plans, review discharge pain management plan, and solicit any patient post-discharge concerns about pain and opioid management. The PA was supervised by a pain physician-psychiatrist (pain and opioid issues), a family physician (primary care implementation issues), and a trauma surgeon (issues related to infection or other trauma complications).

The PA offered support *as needed* at weeks 1, 2, 4, 8, 12, 16, and 20 after discharge or until the PCP office indicated they no longer needed support or the patient had tapered off opioids or were no longer following up with their PCP. If no PCP was identified by the patient, the PA made an effort to identify a PCP for the patient. If the patient identified a PCP with whom they planned to follow-up, the PA called the PCP’s office to describe the study, determine if the patient had a follow-up appointment, and review the discharge instructions. If the PCP was unavailable, the PA asked to speak with other clinical staff (e.g., registered nurse or medical assistant) or clerical staff, describing the study and its purpose of providing collaborative support for pain and opioid taper following a trauma hospitalization.

Support offered by the interventionist to the PCP included:

1. Faxing the patient’s discharge summary, discharge instructions, and a detailed study instruction sheet to the PCP within a few days of the patient’s discharge.
2. Contacting the hospital trauma team if questions about trauma recovery arose.
3. Advising on the opioid taper plan if it was not proceeding as planned, including any concerns about prescription opioid use, misuse, or abuse or illicit opioid use.
4. Problem solving if the PCP had any concerns about their patient’s pain management.
5. Arranging a case presentation to a multidisciplinary telemedicine pain specialist panel about the patient if the PCP desired additional advice.

### Usual care

Patients randomized to usual care received standard hospital discharge instructions and a written information on managing opioid medications after discharge. No other alterations or restrictions in usual follow-up care were imposed.

### Measures

#### Descriptive measures

At the time of study enrollment, the following information was collected from the electronic medical record: age, sex, language preference, ZIP code, Injury Severity Score, Glasgow Coma Score, injury locations, hospital days, opioid exposure inpatient pain management strategies (total opioid days, IV opioid days, oral opioid days), and admission alcohol and drug screens. The following pain, opioid and substance use information was collected: (a) pre-admission chronic pain, (b) lifetime opioid exposure, opioid exposure in pre-trauma month, (c) lifetime cannabis, past year non-medical drug use.

### Primary outcomes

The primary outcomes of this randomized trial were pain and opioid use in the post-discharge period. The primary pain outcome was the Total Pain, Enjoyment of life, and General activities (PEG) score. The PEG is a three item self-reported assessment of average pain intensity (P), interference with enjoyment of life (E), and interference with general activity (G). Construct validity of the PEG is good for various pain-specific measures and comparable to that of the legacy Brief Pain Inventory (BPI). The PEG has been demonstrated to be sensitive to change and be able to differentiate between patients with and without pain improvement at 6 months [10]. 

The primary opioid outcomes were mean daily prescribed opioid dose in oral morphine equivalent dose (MED) milligrams (continuous outcome), and percent at or below self-reported baseline pre-trauma opioid dose (categorical outcome). These measures were collected through the electronic medical record (EMR) access to Washington State Prescription Drug Monitoring Program (PDMP) data for 12 and 24 week opioid use. These record prescriptions but not actual patient opioid use.

### Secondary outcomes

PROMIS-29 Health Profile (29 items) [11] The PROMIS-29 v2.0 profile assesses pain intensity using a single 0–10 numeric rating item and seven health domains (physical function, fatigue, pain interference, depressive symptoms, anxiety, ability to participate in social roles and activities, and sleep disturbance) using four items per domain. It has been used to monitor health outcomes after trauma [12]. For PROMIS instruments, a score of 50 is the average for the United States general population with a standard deviation of 10. A higher PROMIS T-score represents more of the concept being measured. For negatively-worded concepts like Anxiety, a T-score of 60 is one SD worse than average. By comparison, an Anxiety T-score of 40 is one SD better than average. However, for positively-worded concepts like Physical Function-Mobility, a T-score of 60 is one SD better than average while a T-score of 40 is one SD worse than average.

DAST-10: Drug Abuse Screening Test (10 items) [13] is a self-reported screening tool that assesses patient drug use (including both nonmedical use of drugs and excessive use of prescription drugs) over the 12-month period leading up to the time of the screening, yielding a quantitative index. The DAST-10 total score can range from 0 to 10.

Alcohol Use Disorders Identification Test Screen (3 items) [14] The 3-item AUDIT-C measures alcohol consumption [frequency, quantity, and binge-drinking (defined as ≥ 6 drinks on any one occasion)] during the past six months. It has been used to assess problem drinking in patients with chronic pain [15]. The AUDIT-C is scored on a scale of 0–12 (scores of 0 reflect no alcohol use). In men, a score of 4 or more is considered positive; in women, a score of 3 or more is considered positive. Generally, the higher the AUDIT-C score, the more likely it is that the patient’s drinking is affecting his/her health and safety.

Monitoring the Future cannabis questions (4 items) [16] Monitoring the Future is a NIDA-sponsored survey asking participants to report their drug use behaviors across three time periods: lifetime, past year, and past month. Four items assess recent cannabis use. It has been used to monitor cannabis in patients with chronic pain treated with opioids [17]. 

HUNT3 study patient experience with PCP items (5 items) [18] is a self-report survey concerning satisfaction with PCP care adapted from the Hunt Norwegian Pain Study [19]. We report here only on the satisfaction with pain care item.

### Randomization procedures

Study participants were randomized 1:1 according to computer generated sequence to receive either the opioid taper support intervention or usual care according to a computer-generated randomization list in sealed envelopes. Randomization was initially stratified according to whether the patient was taking regular opioids during the month prior to injury, but this stratification was discontinued due to low overall recruitment related to COVID-19, making it impossible to oversample individuals taking opioids prior to injury.

For this study, the proposed sample size of 100 patients would have provided 80% power to detect a 23% decrease in the proportion of patients on opioids at 6 months, from 30 to 7%, in a z-test with pooled variance and an alpha level of 0.05. For pain outcomes, assuming a final study population of 80 subjects *with independent PCPs*, we would have 80% power to detect a difference in PEG score of 1.3 points between the treatment and control arm, with a standard deviation of 2.1 and alpha level 0.05. These treatment effects are similar to those used to power other pain and opioid trials [20, 21]. Due to recruitment restrictions associated with the COVID-19 epidemic, the trial was stopped after 73 subjects were randomized. This sample would yield 80% power to detect a 13.6% difference in opioid dose and a 2.1 point decrease in PEG score.

### Statistical analyses

All primary and secondary statistical analyses were conducted with the intent-to-treat sample. Continuous dependent variables included: baseline, 3- and 6-month assessments of the PEG scale, opioid dose, and PROMIS-29 scale scores, as well as AUDIT-C alcohol and DAST drug use scores. Dichotomous (any use vs. none) opioid use variables were also analyzed in the pre-trauma month, and at 3- and 6-month timepoints. Continuous depression (PHQ9) scores were obtained only at 3 and 6 months. Baseline demographic, injury characteristics, trauma care interventions and baseline (pre-injury) pain, and substance use variables were compared using chi-square tests for categorical variables or t-tests for continuous variables between intervention and control groups. No variables were found to be imbalanced at baseline.

Mixed effect regression models were fit containing time categories, group (I vs. C) and group by time interactions. Adjusted mean difference or relative risk (aRR) and 95% confidence interval (CI) were derived from the models. All analyses were conducted using SAS Software Version 9.4 (SAS Institute Inc., Cary, NC, USA).

## Results

### Baseline characteristics of the Sample

As can be seen in Fig. 1, of 4738 potentially eligible subjects identified through medical record review, 1105 were eligible and 78 consented to the study and were randomized.

Table 1 shows the sociodemographic characteristics of the study participants, as well as baseline clinical characteristics, substance use, and pain care received during hospitalization. There were no statistically significant differences between the groups randomized to the opioid taper support intervention vs. usual care. Overall, the mean (standard deviation, SD) age of the study participants was 47.0 (17.4) years. The sample was 72% male. According to residence ZIP codes, 59% of the sample lived in an urban area, 27% lived in a large rural town, 11% lived in a small rural town, and 3% lived in an isolated small rural town.

**Table 1:** Sample demographic and clinical characteristics

Characteristic	Intervention group N = 37		Usual care group N = 36		Overall cohort N = 73	
	n	%	n	%	n	%
**Demographics**						
Age, Mean(SD)	45.4	17.3	48.2	17.6	47	17.4
Male	26	72.2	26	72.2	52	72.2
Language preference-English	36	97.3	36	100	72	98.6
Race						
White	35	94.6	31	86.1	66	90.4
African American	2	5.4	1	2.8	3	4.1
American Indian	0	0	2	5.6	2	2.7
Pacific Islander	0	0	2	5.6	2	2.7
Hispanic	1	2.7	2	5.6	3	4.1
Residence location*						
Urban	21	58.3	28	77.8	49	68.1
Large Rural Town	10	27.8	5	13.9	15	20.8
Small Rural Town	4	11.1	1	2.8	5	6.9
Isolated Small Rural Town	1	2.8	2	5.6	3	4.2
**Injury descriptors**						
Injury Severity Score, Mean(SD)	13	8.5	13.5	8	13.2	8.2
**Inpatient pain management strategies**						
Total opioid days, Mean(SD)	5.1	3.2	5.7	3.6	5.4	3.3
Total IV opioid days, Mean(SD)	0.4	0.7	0.6	1.6	0.5	1.2
Total oral opioid days, Mean(SD)	4.7	3	5.1	3.1	4.9	3
**Substance use disorder testing**						
Admission alcohol testing						
Not tested	6	16.2	9	25	15	20.6
Neg	28	75.7	20	55.6	48	65.8
Yes(1-79mg/dl)	2	5.4	3	8.3	5	6.9
Yes(80mg/dl or higher)	1	2.7	4	11.1	5	6.9
Admission urine toxicology screening						
Not tested	25	67.6	20	55.6	45	61.6
Neg	6	16.2	9	25	15	20.6
Pos	6	16.2	7	19.4	13	17.8
Amphetamine/Methamphetamine	2	5.4	2	5.6	4	5.5
Cocaine	0	0	1	2.8	1	1.4
Cannabis	4	10.8	6	16.7	10	13.7
**Inpatient stay characteristics**						
Total Hospital Days, Mean (SD)	4.5	3.4	5	3.8	4.7	3.6
Opioid daily dose at hospital discharge, MED Mean(SD)	75.3	28	66.7	25.2	71	26.8
**Pre-trauma pain, opioid, drug use**						
Chronic pain for the last 3 months pre-trauma	15	40.5	11	32.4	26	36.6
Lifetime Opioid exposure	28	75.7	26	76.5	54	76.1
Opioid exposure (N) during pre-trauma month (from WA PDMP)	3	8.3	4	11.1	7	9.7
Opioid dose (mean MED) during pre-trauma month (from WA PDMP)	1.8	6.3	5.3	18.3	3.5	13.7
Lifetime Cannabis (# times used)						
0	10	27	10	29.4	20	28.2
1–2	5	13.5	2	5.9	7	9.9
3–5	1	2.7	2	5.9	3	4.2
10–19	2	5.4	6	17.7	8	11.3
20–39	2	5.4	2	5.9	4	5.6
40 or more	17	46	12	35.3	29	40.9
Use of any drug other than required for medical reason in 12 months prior to trauma	1	2.7	2	5.7	3	4.2

*Residence location is defined using Rural Urban Commuting Area Codes linked to the ZIP code of the patient’s residence. ^34, 35^

Some percentages do not add up to a total of 100% due to rounding error

WA PDMP = Washington State Prescription Drug Monitoring Program

During the month prior to admission, 37% reported experiencing chronic pain. During their lifetime, 76% had been prescribed opioids. During the month prior to their trauma, 10% had received opioids. The mean (SD) Injury Severity Score was 13.2 (8.2) and 85% (*N* = 62) of the sample had an admission Glasgow Coma Score of 15. On admission alcohol testing, 21% were not tested, 66% were negative, 7% were positive but below the legal limit for driving, and 7% were above the legal limit. On admission urine toxicology screen, 62% were not tested, 21% tested negative, 14% tested positive for cannabis, 6% positive for amphetamines, and 1% for cocaine. Over their lifetime, 41% of the sample reported using cannabis 40 or more times.

Mean hospital stay was 4.7 (SD = 3.6) days, with 29% spending some time in intensive care. During hospitalization 75% had orthopedic procedures and 7% had been intubated at some point.

### Intervention delivered

PCP offices were successfully contacted on behalf of 19 of the 37 intervention patients, and they had a total of 36 consults. For patients without a PCP, an attempt was made to schedule a follow-up appointment with a new PCP at their local Federally Qualified Health Center (FQHC), but this was not successful, either due to patient lack of interest or lack of availability of clinicians at the FQHC. Of these 36 consults, 20 (56%) involved a medical assistant, 8 (22%) involved the PCP and 8 (22%) involved a nurse. Pain management was discussed in 68% of these consults and opioid management was discussed in 79%.

### Primary and secondary outcomes at 3 and 6 months

Table 2 shows the observed values for the primary and secondary outcome measures in each group at baseline (prior to hospital discharge) and 3 and 6 months after discharge, as well as the differences between the intervention and usual care groups in the mean change from baseline to 3 and 6 months.

**Table 2:** Primary and secondary patient outcomes

	Baseline- at discharge		3 months		6 months			3 m-baseline		6 m-baseline
	I, Mean(SD)	UC, Mean (SD)	I, Mean (SD)	UC, Mean (SD)	I, Mean (SD)	UC, Mean (SD)		I vs. UC, diff.		I vs. UC, diff. (95% CI)
Pain severity	3.7(3.4)	4.1(3.7)	3.7(2.5)	2.9(2.1)	3.6(2.6)	2.7(2.2)		1.3(-0.2,2.9)		1.2(-0.3,2.8)
Enjoyment of life interference	3.3(3.8)	3.9(3.9)	3.9(3.2)	3.7(2.9)	3.6(3.3)	3.0(3.1)		1.2(-0.6,3.0)		1.3(-0.5,3.1)
General activity interference	3.2(4.0)	3.9(4.1)	4.0(3.3)	4.2(3.1)	3.9(3.4)	3.2(3.0)		0.8(-1.0,2.7)		1.3(-0.6,3.1)
PEG mean score	3.5(3.6)	3.9(3.6)	3.9(2.8)	3.6(2.6)	3.7(3.0)	3.0(2.7)		1.1(-0.5,2.7)		1.2(-0.4,2.8)
Post-trauma opioid use										
Any Opioid use n(%)	37 (100)	36(100)	6(16.7)	3(8.8)	7(19.4)	8(23.5)		2.55(0.61,10.75)		1.12(0.29,4.37)
Group mean daily opioid dose (MED)	75.3(28.0)	66.7(25.2)	8.6(27.9)	2.1(8.5)	5.2(17.4)	2.0(6.7)		-1.8(-13.7,10.0)		-5.4(-17.6,6.9)
PROMIS- 29 scale scores										
Physical Function	48.9(11.3)	44.5(14.8)	36.3(8.3)	39.2(9.4)	41.6(8.8)	44.6(9.8)		-7.5(-13.1,-1.8)*		-6.9(-12.5,-1.3)*
Anxiety	50.6(10.9)	52.4(12.1)	50.1(9.4)	51.9(10.4)	51.2(9.6)	49.6(11.2)		1.1(-3.6,5.9)		3.5(-1.2,8.1)
Depression	46.2(7.4)	48.7(9.1)	49.3(9.7)	52.6(11.2)	48.9(10.2)	50.5(12.7)		-0.6(-5.9,4.7)		0.7(-4.5,5.9)
Fatigue	45.8(11.3)	48.4(11.4)	50.0(10.5)	49.6(10.8)	49.8(10.8)	47.0(11.0)		3.9(-1.9,9.6)		5.2(-0.4,10.8)
Sleep Disturbance	49.1(10.6)	51.3(9.6)	51.4(10.5)	52.1(8.6)	50.2(10.4)	48.9(8.8)		2.8(-2.4,7.9)		4.2(-0.9,9.2)
Ability to participate in social roles	56.2(10.7)	53.4(12.9)	42.1(8.6)	44.6(10.3)	47.0(9.0)	49.2(11.9)		-5.0(-11.3,1.2)		-4.9(-11.0,1.2)
Pain interference	53.4(11.4)	56.1(13.6)	57.7(10.5)	58.0(10.8)	58.7(8.4)	54.4(10.1)		3.3(-2.7,9.2)		7.1(1.2,12.9)*
Pain intensity	3.8(3.0)	3.8(3.4)	3.7(2.7)	3.1(2.2)	3.7(2.9)	2.6(2.2)		0.7(-0.7,2.2)		0.9(-0.5,2.3)
AUDIT-C, past year	3.3(2.3)	3.5(2.7)	3.0(1.9)	3.3(3.1)	2.7(2.5)	2.9(3.2)		0.04(-0.9,0.9)		0.3(-0.6,1.2)
DAST total, past year	0.1(0.3)	0.2(1.0)	0.0(0.0)	0.5(1.7)	0.1(0.4)	0.3(1.4)		-0.4(-1.0,0.2)		-0.03(-0.7,0.6)

Satisfaction with pain care xx xx 9.5(1.0) 9.7(0.7) 8.8(1.8) 9.5(1.3)

*p < 0.05; +at hospital discharge

*At 3 months*, mean total PEG scores were similar in the intervention and usual care groups with no significant differences between the groups. There were also no significant differences between the intervention and usual care groups in the change in overall PEG score or in the individual components of the PEG score (Pain severity, Enjoyment of life interference, General activity interference) between baseline and 3 months.

All participants were taking opioids when discharged from the hospital (as required by study inclusion criteria) with a mean dose of 75 mg MED in the intervention group and 67 mg MED in the usual care group (Table 3). By 3 months, 6 patients (17%) were prescribed opioids in the intervention group and 3 patients (9%) in the usual care group. Mean daily opioid dose (MED) for the overall randomized groups at 3 months was 8.6 (SD = 27.9) in the intervention group and 2.1 (SD = 8.5) in the usual care group. In the intervention group, 86% were at or below their pre-trauma opioid dose, compared to 94% of the usual care group. Opioid dose at 3 months was reduced from hospital discharge (baseline) in both the intervention and usual care groups, with no significant difference between groups in dose reduction (adjusted mean difference between 3 months and baseline = -1.81 mg MED; 95% CI: -13.65, 10.03; *p* = 0.76) or in percent with reduction from baseline to 3 months in dose (mean, 89.6% vs. 93.7%; adjusted mean difference between 3 months and baseline = 4.1%; 95% CI: -9.4%, 17.7%; *p* = 0.54; data not shown). Data are based on opioids prescribed, rather than opioids actually used by patients.

**Table 3:** Opioid use patterns

	Time			
	Pre-trauma	Baseline (discharge)	3 months	6 months
**Intervention**				
Using opioids, n(%)	3(8.3)	37(100)	6(16.7)	7(19.4)
Mean daily opioid dose (MED) among those using opioids, Mean(SD)	21.7(7.6)	75.3(28.0)	60.4(51.9)	42.7(33.1)
Mean daily opioid dose (MED) for intervention group overall, Mean(SD)	1.8(6.3)	75.3(28.0)	8.6(27.9)	5.2(17.4)
**Usual Care**				
Using opioids, n(%)	4(11.1)	36(100)	3(8.8)	8(23.5)
Mean daily opioid dose (MED) among those using opioids, Mean(SD)	47.4(35.1)	66.7(25.2)	24.1(20.1)	19.5(10.8)
Mean daily opioid dose (MED) for usual care group overall, Mean(SD)	5.3(18.3)	66.7(25.2)	2.1(8.5)	2.0(6.7)

There were statistically significant differences between the intervention and usual care groups when comparing 3 month to baseline PROMIS-29 scale score changes. The two significant changes were physical function and pain interference. Physical function declined in both groups but the decline was less in the usual care group. (-7.5 mean difference between groups) and pain interference. There were no differences between the intervention and usual care groups when comparing 3-month to baseline AUDIT-C and DAST-10 score changes. At 3 months, PHQ-9 depression score in the collaborative care group was 4.8 (SD = 4.3), while in the usual care group it was 7.3 (SD = 6.6), *p* = 0.10. Satisfaction with pain care score in the collaborative care group was 9.5 (SD = 1.0), which in the usual care group it was 9.7 (SD = 0.7), *p* = 0.47.

*At 6 months*, mean total PEG scores were similar in intervention and usual care groups with no significant differences between groups. There were also no significant differences between the intervention and usual care groups in the change in overall PEG score or in the individual components of the PEG score (Pain severity, Enjoyment of life interference, General activity interference) between baseline and 6 months.

At 6 months, 7 patients (19%) were on opioids in the intervention group and 8 patients (23%) in the usual care group. Mean daily opioid dose (MED) was reduced from hospital discharge (baseline) in both the intervention and usual care groups, with no significant difference between groups in dose reduction (adjusted mean difference between 6 months and baseline = -5.35 mg MED; 95% CI: -17.60, 6.90; *p* = 0.39) or in percent with reduction from baseline to 6 months in dose (mean, 93.7% vs. 92.8%; adjusted mean difference between 6 months and baseline = -0.9%; 95% CI: -14.5%, 12.7%; *p* = 0.89; data not shown).

At 6 months, there were no statistically significant differences between the intervention and usual care groups when comparing 6 month to baseline PROMIS-29 scale scores except for small improvements in physical function and pain interference, favoring the usual care group. There were no differences between the intervention and usual care group when comparing 6-month to baseline AUDIT-C and DAST-10 score changes There were no differences between the intervention and usual care group when comparing 6-month to baseline AUDIT-C and DAST-10 score changes. At 6 months, thePHQ-9 score in the collaborative care group was 5.2 (SD = 5.7), while in the usual care group it was 6.1 (SD = 6.9) *p* = 0.55. Satisfaction with pain care score in the collaborative care group was 8.8 (SD = 1.8), which in the usual care group it was 9.5 (SD = 1.3), *p* = 0.04 (data not shown).

**Fig. 3:**
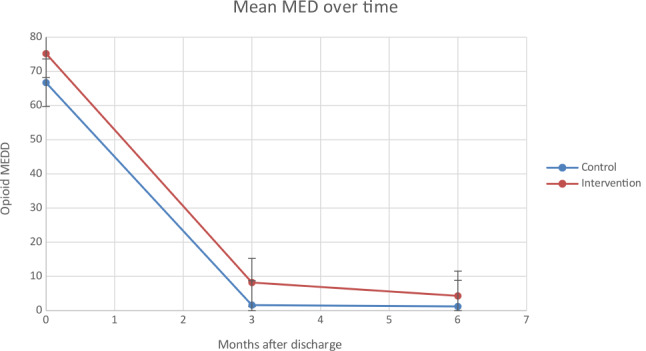
Mean opioid dose (MED) over time, intervention vs. control groups

Figure 3 displays the PEG mean score over time, comparing intervention vs. control groups at baseline, 3 and 6 months. Figure 4 displays the mean daily opioid dose (in morphine equivalent dose) over time, comparing intervention vs. control groups at baseline, 3 and 6 months.

## Discussion

Our randomized trial of an opioid taper support intervention failed to improve pain or opioid outcomes at 3 and 6 months compared with usual care. Pain, as assessed by the 3-item PEG score, was not significantly different between intervention and control groups. There were no significant differences in continuous or categorical measures of opioid dosing. At the same time, our trial found that the majority of our generally lower risk patients hospitalized for trauma successfully tapered off of opioids post-hospitalization regardless of whether their PCP had access to additional support or whether they had a PCP at all. We are unaware of other randomized trials of post-discharge PCP support to improve pain and opioid outcomes in trauma patients. Trials of inpatient opioid-sparing treatment regimens have shown decreased rates of opioid misuse after discharge [22]. Future research on improving pain and opioid outcomes after trauma hospitalization will need to focus on interventions both preceding and following discharge.

There are multiple reasons that might explain why our trial intervention did not produce significant differences. First, we randomized 73 rather than 80 patients with independent PCPs due to recruitment problems related to COVID-19, so our trial was underpowered for our primary opioid and pain outcomes. Although trauma patients continued to be admitted to our study hospital throughout COVID, many hospital policies about access to patients for research, admission and discharge policies, and the capacity of the hospital to provide follow-up were altered. Second, both the intervention and control groups had low rates of opioid use and relatively low pain scores at 3 and 6 months. We aimed to recruit 50% “high-risk” patients with opioid use prior to their trauma for our sample, but we were able to recruit < 10% with ongoing opioid use, likely due to COVID-19-related constraints on hospital admissions and overall subject recruitment, making it impossible to oversample individuals using opioids at the time of their trauma. Our sample was therefore mostly lower risk patients who were not opioid tolerant at the time of their injury. This meant that our intervention did not have much opportunity for improvement in this lower risk sample. Nevertheless, the number of patients with any opioid use rose slightly from 3 to 6 months (from 6 to 7 in the intervention arm, and from 3 to 8 in the usual care arm. The reason for this rise is unclear. Third, COVID-19 limited in-person engagement with our research participants. We had planned an initial in-person meeting between our interventionist and research participants randomized to our support intervention, but all contact with our physician assistant was conducted over the phone, limiting development of rapport and collaboration.

An unanticipated finding that may have influenced our study outcomes is that we were unable to identify an established PCP for almost half of our research participants. Our study population was largely young and male, the group least likely to have an established PCP. Since the intervention was largely focused on the PCP, the study did not have the opportunity to implement the intervention as intended for a substantial proportion of patients. Of the 19 (51%) intervention subjects with confirmed and contacted PCP offices, only 22% involved direct interaction between the physician assistant and the PCP. Many PCPs were preoccupied with COVID-19 care at the time of the study. Nevertheless, PCPs responding to a post-intervention survey reported that the program was acceptable, appropriate, and feasible [23]. The study’s limited contact with PCPs attenuated the strength of the intended intervention. Any future study should anticipate these problems in establishing collaboration with PCPs. More detailed procedures for assisting trauma patients in establishing care with a local PCP and for establishing communication with that PCP are needed.

Two final methodological issues are important to note. First, although we screened 4738 hospital inpatients for our study, we consented only 78. This was due not only to the COVID-19 epidemic, but to the many inclusion and exclusion criteria of our pilot study. We sought to focus on patients from rural Washington areas with moderate to severe trauma discharged on opioids who did not have serious head trauma, Opioid Use Disorder, or unstable psychiatric disorders. This focus clearly limited the scope of our study, and future studies may want to reduce these exclusions. Second, a number of our outcome measures (PEG scale, PROMIS scales) are designed to be used in outpatient care. The baseline values for our outcome analyses were collected while our participants were still hospitalized, making change scores on these measures difficult to interpret. We do provide unadjusted analyses of scores between groups at 3 and 6 months that are not affected by this problem, however.

As we look forward to future research in this area, we should recognize that collaboration between Level 1 trauma centers and rural primary care is underdeveloped. Communication between advanced trauma and PCPs is not listed among the World Health Organization essentials of trauma care [24], and what little discussion of such collaboration does exist focuses on the roles and needs of acute trauma care providers as they receive rural patients, rather than consideration of the needs of PCPs and the discharged trauma patients themselves [25]. 

There remains a need for improved pain care of patients who are discharged from trauma units. Trauma prompts 2.3 million hospitalizations a year. Opioid use for > 90 days after injury in the US in 2009–2012 was 15%.[26] Opioid use 3 to 4 months after trauma-related orthopedic surgery ranges from 20–35%.[27] Recent legislation in Washington State that encourages limited amounts and duration of discharge opioids may have reduced risks for prolonged opioid use in our sample [28]. National implementation of the 2016 CDC Opioid Guidelines may have also played a role [29]. Nevertheless, the generally favorable opioid outcomes in both our intervention and control groups suggests that the risks of opioid misuse after discharge may be concentrated in the higher risk patients who are opioid tolerant or have a history of SUD [30]. Future interventions may best be focused on this population.

Any program to address post-trauma opioid risks must also address post-trauma pain care. Collaborative care models have been adapted for chronic pain care, but not post-trauma pain care. Collaborative care models using care managers to improve chronic illness care have been adapted for collaborative care of chronic pain, and shown efficacy in randomized clinical trials [31]. Collaborative care for chronic pain delivered over the phone has been shown effective in a randomized trial [32]. Future trials of collaborative opioid taper support interventions will need to address: the lack of established relationships with a PCP among many patients recovering from trauma, the many other acute and chronic disease issues that these PCPs must manage on a daily basis, and the lack of capacity at Level 1 trauma centers to closely follow and monitor patients who have been discharged on opioids.

### Electronic supplementary material

Below is the link to the electronic supplementary material.

Supplementary Material 1

## Data Availability

The dataset supporting the conclusions of this article are available in the Harborview Injury Prevention and Research Center repository hiprc@uw.edu. De-identifed data available with signed data use agreement upon request from Mark Sullivan, sullimar@uw.edu.

## Abbreviations

COTAT: Collaborative Opioid Taper After Trauma
PA: Physician assistant
PCP: Primary care provider
PEG: Pain, enjoyment of life, general activities assessment tool
COVID-19: Corona virus 2019
CDC: Centers for Disease Control and Prevention
OUD: Opioid Use Disorder
RCT: Randomized controlled trial
EMR: Electronic medical record
PDMP: Prescription drug monitoring program
PROMIS-29: Patient Reported Outcome Measurement Information System
DAST-10: Drug Abuse Screening Test
AUDIT-C: Alcohol Use Disorders Identification Test
HUNT-3: Hunt Norwegian Pain Study patient satisfaction tool
PHQ-9: Patient Health Questionnaire-9
SD: Standard deviation
MED: Morphine equivalent dose
FQHC: Federally Qualified Health Center

## References

[1] U.S. Overdose Deaths Decrease in 2023, First Time Since 2018. US Government. 2024. (Accessed May 28, 2024, 2024.

[2] Enns BKE, Thomson T, Dale LM, Min JE, Nosyk B (2021). Opioid analgesic prescribing for opioid-naïve individuals prior to identification of opioid use disorder in British Columbia. Can Addict.

[3] Smolina KCA, Chong M, Zhao B, Park M, Mill C, Schütz CG (2019). Patterns and history of prescription drug use among opioid-related drug overdose cases in British Columbia, Canada, 2015–2016. Drug Alcohol Depend.

[4] Lazaridou APM, Zgierska AE, Garland EL, Edwards RR (2022). Exploring the Relationship between Endogenous Pain Modulation, Pain Intensity, and Depression in patients using opioids for chronic low back Pain. Clin J Pain.

[5] Jamison RNER, Brown R, Barrett BP, Burzinski CA, Lennon RP, Nakamura Y, Schiefelbein T, Garland EL, Zgierska AE (2023). Risk factors for self-harm ideation among persons treated with opioids for chronic low back Pain. Clin J Pain.

[6] Kind SOJ. The Interaction between Chronic Pain and PTSD. Curr Pain Headache Rep; 2019. 10.1007/s11916-019-0828-3 31781875

[7] Waljee JFCD, Steiger RM, Zhong L, Englesbe MJ, Brummett CM (2017). Effect of preoperative opioid exposure on Healthcare Utilization and expenditures following elective abdominal surgery. Ann Surg.

[8] Cortez ARFC, Levinsky NC, Kassam AF, Wima K, Jung AD, Rafferty JF, Paquette IM (2019). The impact of preoperative opioid use on outcomes after elective colorectal surgery: a propensity-matched comparison study. Surgery.

[9] Flynn HKMD, Hsu YJ, Xie A, Shechter R, Hanna M, Speed TJ (2022). A multidisciplinary transitional pain service to improve pain outcomes following trauma surgery: a preliminary report. Scand J Pain.

[10] Krebs EE, Lorenz KA, Bair MJ (2009). Development and initial validation of the PEG, a three-item scale assessing pain intensity and interference. J Gen Intern Med.

[11] Hays RDSK, Schalet BD, Cella D (2018). PROMIS®-29 v2.0 profile physical and mental health summary scores. Qual Life Res.

[12] Hatchimonji JSKE, Chreiman K, Stoecker JB, Reilly PM, Smith BP, Holena DN, Seamon MJ (2021). Beyond morbidity and mortality: the practicality of measuring patient-reported outcomes in trauma. Injury.

[13] Skinner H (1982). The drug abuse screening test. Addict Behav.

[14] Higgins-Biddle JCBT (2018). A review of the Alcohol Use disorders Identification Test (AUDIT), AUDIT-C, and USAUDIT for screening in the United States: past issues and future directions. Am J Drug Alcohol Abuse.

[15] Davis AKWM, Bohnert KM, Bourque C, Ilgen MA (2018). Factors associated with alcohol consumption among medical cannabis patients with chronic pain. Addict Behav.

[16] Parker MAAJ (2018). A prospective study of newly incident cannabis use and cannabis risk perceptions: results from the United States Monitoring the future study. Drug Alcohol Depend.

[17] DiBenedetto DJWV, Wawrzyniak KM, Finkelman M, Paolini J, Schatman ME, Herrera D, Kulich RJ (2018). The Association between Cannabis Use and aberrant behaviors during Chronic Opioid Therapy for Chronic Pain. Pain Med.

[18] Ruan XKA (2016). Consumption of and satisfaction with health care among opioid users with chronic non-malignant pain. Acta Anaesthesiol Scand.

[19] Nordstoga AL, Nilsen TIL, Vasseljen O, Unsgaard-Tondel M, Mork PJ (2017). The influence of multisite pain and psychological comorbidity on prognosis of chronic low back pain: longitudinal data from the Norwegian HUNT study. BMJ Open.

[20] Krebs EEBW, Nelson D, DeRonne BM, Nugent S, Jensen AC, Amundson EC, Manuel JK, Borsari B, Kats AM, Seal KH. Design, methods, and recruitment outcomes of the Veterans’ Pain Care Organizational Improvement Comparative Effectiveness (VOICE) study. Contemp Clin Trials 2023;124. 10.1016/j.cct.2022.107001 36384218

[21] Krebs EECB, Nugent S, Jensen AC, Martinson BC, Goldsmith ES, Donaldson MT, Frank JW, Rutks I, Noorbaloochi S. The evaluating prescription opioid changes in veterans (EPOCH) study: design, survey response, and baseline characteristics. PLoS ONE 2020;15. 10.1371/journal.pone.0230751 PMC7176145 32320421

[22] de Dios CSR, Green C, Klugh JM, Harvin JA, Webber HE, Schmitz JM, Lane SD, Yoon JH, Heads A, Motley K, Stotts A (2023). An opioid-minimizing multimodal pain regimen reduces opioid exposure and pain in trauma-injured patients at high risk for opioid misuse: secondary analysis from the mast trial. Surgery.

[23] Baldwin LMKL, Sullivan MD, Gordon DB, James A, Tauben DJ, Arbabi S. Lessons from the implementation of a trauma center-based program to support primary care providers in managing opioids and pain after trauma hospitalization. Trauma Surgery and Acute Care Open. 2023;in press. 10.1136/tsaco-2022-001038 PMC9944266 36844370

[24] Guidelines for essential trauma care. World Health Organization. 2012. (Accessed 12-6-2022, 2022.

[25] Keeves JEC, Beck B, Gabbe BJ (2019). The relationship between geographic location and outcomes following injury: a scoping review. Injury.

[26] Alghnam SCR (2017). Traumatic injuries and persistent opioid use in the USA: findings from a nationally representative survey. Inj Prev.

[27] Rosenbloom BNMC, Canzian S, Kreder HJ, Katz J (2017). Predictors of prescription opioid use 4 months after traumatic Musculoskeletal Injury and corrective surgery: a prospective study. J Pain.

[28] HB 1427-2017-18. State of Washington. 2018. (Accessed May 28, 2024, 2024.

[29] Sutherland TNWH, Pinto R, Newcomb C, Brensinger C, Gaskins L, Bateman BT, Neuman MD (2021). Association of the 2016 US Centers for Disease Control and Prevention Opioid Prescribing Guideline with changes in Opioid dispensing after surgery. JAMA Netw Open.

[30] Kim YCA, Wima K (2018). Impact of preoperative opioid use after emergency general surgery. J Gastrointest Surg.

[31] Dobscha SK, Corson K, Perrin NA (2009). Collaborative care for chronic pain in primary care: a cluster randomized trial. JAMA.

[32] Kroenke K, Krebs EE, Wu J, Yu Z, Chumbler NR, Bair MJ (2014). Telecare collaborative management of chronic pain in primary care: a randomized clinical trial. JAMA.

